# Editorial: New therapies in the treatment of sarcomas

**DOI:** 10.3389/fendo.2023.1137736

**Published:** 2023-01-18

**Authors:** Alison Gartland, Michela Pasello, Frédéric Lézot, Francois Lamoureux

**Affiliations:** ^1^ Department of Oncology and Metabolism, The University of Sheffield, Sheffield, United Kingdom; ^2^ Experimental Oncology Laboratory, IRCCS Istituto Ortopedico Rizzoli, Bologna, Italy; ^3^ Laboratory of childhood genetic diseases, Sorbonne University, INSERM UMR933, Hospital Armand Trousseau AP-HP, Paris, France; ^4^ INSERM 1307 CNRS 6075 CRCI2NA - Team 9, CHILD - Faculty of medicine, Nantes University, Nantes, France

**Keywords:** primary bone tumors, osteosarcoma, Ewing sarcoma, resistance to treatment, bone microenvironment, clinical analysis

Bone sarcomas, especially osteosarcoma and Ewing sarcoma, are primary bone tumors mainly affecting children and young adults (1–3). Current treatment has not progressed for the last 40 years and is based on a multimodal therapy including neo-adjuvant chemotherapy, surgical resection, and radiotherapy in some cases (1, 4). With these therapeutic options, the survival rate reaches 60-70% for the localized forms but drastically drops down to 25-30% for the poor responders to treatment or for patients with metastasis at the diagnosis (2, 4). The unsatisfactory outcomes and side effects associated with the current chemotherapy underscore the urgent need to find new therapeutic approaches for osteosarcoma and Ewing sarcoma. In this context, a better knowledge of bone biology has become essential for the medical and cancer research communities.

The aim of the present Research Topic was to assemble manuscripts addressing the questions relating to primary bone cancers development including genomic and proteomic analyses, clinical analyses, as well as future directions for new therapies and clinical trials for these pediatric tumors. The topic was more precisely open to: (i) epigenomic, genomic and proteomic analysis in the development of primary bone tumors, (ii) cellular and molecular mechanisms underlying primary bone tumors, (iii) clinical analysis (histopathology and imaging) in primary bone tumors, (iv) importance of bone microenvironment (bone cells, immune cells, fibroblasts and so on) to support development and progression of primary bone tumors, and (v) resistance to treatment and future horizons in treatments of primary bone tumors.

As editors of this Research Topic, we had the pleasure of reading various very interesting articles in the field. We summarized the main findings and perspectives detailed in each accepted article in this editorial. This Research Topic assembles four original research manuscripts, two case report manuscripts and one brief research report manuscript.

The original research manuscripts are all relative to osteosarcoma, with two concerned with the immune escape of osteosarcoma. A study by Sung et al. evidenced that interferon (IFN) consensus sequence binding protein (ICSBP), a transcription factor induced by IFN-γ, increases expression of the immune check point PD-L1 (programmed death-ligand 1) in osteosarcoma cells favoring the immune escape of osteosarcoma. Interestingly, PD-L1 knockdown was shown to decrease growth of tumor xenografts and increase doxorubicin sensitivity of ICSBP-overexpressing osteosarcoma. The second study by Zhang et al. investigated whether ferroptosis-related long non-coding RNAs (FRLs) expressed by the tumor could be used to predict prognosis and immune characteristics in osteosarcoma patients. Through the screening of 43 prognosis-related FRLs, the authors identified two molecular subtypes with different clinical outcomes. They then defined an FRL prognostic signature for osteosarcoma called “FRL-score”, which could distinguish immune function, immune score, stromal score, tumor purity, and tumor infiltration of immune cells in different osteosarcoma patients.

The third original research manuscript by Hong et al. is a retrospective evaluation of the benefit of high-dose chemotherapy (melphalan, etoposide and carboplatin) as a second line treatment for patients with non-metastatic osteosarcoma and <90% necrosis (poor responder) after the first-line neoadjuvant chemotherapy (mainly cisplatin, doxorubicin and methotrexate), in a cohort of patients with osteosarcoma treated at the Seoul National University Children’s Hospital from 2000 to 2020. This evaluation clearly evidences that high-dose chemotherapy increases the favorable outcomes in the poor responder patients.

The fourth original research manuscript by Morice et al. concerns the pro-metastatic effect of YAP-driven lung metastatic development in osteosarcoma and more specifically the implicated subjacent molecular mechanisms. Based on both *in vitro* and *in vivo* experiments, the authors elegantly demonstrate that YAP interacts with Smad3 and stimulates the transcriptional activity of TGF-β/Smad3, thereby enhancing the ability of TGF-β to stimulate lung metastases development.

The two case report manuscripts by He et al. and Yang et al. present interesting therapeutic options for the management of two rare sarcomas, respectively the soft tissue sarcoma of adolescent with an YWHAE-NUTM2B Fusion and the primitive myxoid mesenchymal tumor of infancy. In the first case, the combined therapy of Epirubicin and Anlotinib demonstrates a remarkable efficiency on the primary tumor as on the lung metastatic nodules (He et al.). In the second case, the intratumoral injection of bleomycin substantially helps in developing better surgical conditions or improving outcomes in non-surgical patients (Yang et al.).

Last but not least, the brief research report by Han et al., which also concerns a rare type of sarcoma namely the infantile fibrosarcoma, underlines the complexity of the management of such tumor mainly due to the very small number of feedback from clinical services simply linked to the rarity of these tumors. Based on a retrospective analysis of a remarkable cohort of eleven patients, the authors propose a useful guide for the management of patients with infantile fibrosarcoma, taking into account the different grades of the disease.

The guest Editors want to thank all the authors for sharing their highly interesting results in this topic and all the Reviewers that kingly give their time and made this Research Topic possible.

## Author contributions

All authors listed have made a substantial, direct, and intellectual contribution to the work and approved it for publication.
